# The President of the French Republic at the French Hospital

**Published:** 1903-07-11

**Authors:** 


### THE PRESIDENT OF THE FRENCH REPUBLIC AT THE FRENCH HOSPITAL.

On Tuesday morning the President of the French Republic paid a visit to the French Hospital in Shaftesbury Avenue. The time fixed for the reception was a quarter past nine, and soon after half past eight a number of invited guests, consisting entirely of the male sex, had assembled in the prettily-decorated committee room. The approach of the distinguished visitor was heralded in the distance by the cheers of his countrymen and others who had assembled at that early hour in order to have the pleasure of greeting M. Loubet. Just before the carriage arrived there was a sudden silence which was afterwards explained by the fact that a slight accident had happened, and the onlookers were so concerned that they ceased cheering for the moment. The travelling escort of the President was formed from the 2nd Life Guards, and as the riders pulled up their horses opposite the hospital, two of the animals fell, owing to the slippery state of the road. One, Lieutenant Ashton, quickly regained his seat, but the other, Corporal Stowell, unfortunately, did not escape so lightly, and had his foot wrenched through getting it caught in the stirrup. He was attended to in the hospital, and afterwards taken to the Knightsbridge Barracks by the R.M.O. in the senior surgeon's carriage. The President kindly inquired after him, and shook hands with him upon hearing that he was not much hurt.

On entering the hospital, the President, who was accompanied by M. Cambon, the French Ambassador, and Lord Howe, Lord in Waiting, was received by the members of the committee, the honorary treasurer, M. Ernest Lazarus, the honorary secretary M. Ernest Rüffer, the "econome honoraire" M. Eugène Cocquerel, the members of the medical staff, including Dr. Achille Vintras, chief physician, Mr. Edmund Owen, senior surgeon, and Dr. J. Barlet, resident medical officer, with the following members of the committee, Messieurs Arthur Baume, Venant Benoist, Alexander Casella, Léon Clerc, E. A. Hambro, A. Hernu, E. Karminski, E. Roehrich, and Albert Sauvée. Dr. Vintras, having introduced the members of the Committee and medical staff individually to the President, read the following address:—

### The Address.

To His Excellency Monsieur Emile Loubet, President of the French Republic.

Monsieur le Président,—The short time of your stay in England has not removed from your mind the touching thought of visiting the French Hospital. The committee of management, the medical staff, and in fact the whole *personnel* have been deeply touched by it, and I am happy, Sir, to offer to you, in their name and in my own, the most profound devotion towards yourself and the French Government. We shall always remember with gratitude that the Government of the Republic wished to be placed at the top of the subscription list for the construction of the building in which we have the honour to receive you to-day. A generous Frenchman, Monsieur Anchois, bequeathed 15 years ago the magnificent sum of £72,000, thus assuring in a great measure the future of the hospital, and of which sum we receive the interest through the Minister of Foreign Affairs. This legacy has enabled us to build at the seaside a convalescent home and retreat for old French people. The Government, recognising the great usefulness of this home attached to the hospital, has very generously given a superb donation of £8,000 to help towards its Building Fund. The great success of the bazaar which took place last year at the French Embassy was the crowning glory of M. Paul Cambon's indefatigable efforts to ameliorate the position of the French charities in London. From the receipts of this bazaar the Ambassador gave to the hospital the sum of £1,000 to help to pay the debt of the Building Fund, and also a further sum of £500 for the construction of a pavilion for the preventive treatment of consumption. May we ask you, Monsieur le Président, to be good enough to go over the hospital? We have tried our best to make it worthy of France, and the first condition for admission here is to be able to speak our language. Poverty, sickness, and exile from one's native land are amongst the most cruel calamities that can occur, and it is to help this triple misfortune that the hospital was founded. To help the needs of these unfortunate people, to relieve their sufferings, and to give them back, so to speak, their native land by surrounding them with people who speak their language, that is the mission we have undertaken. We thank you, Monsieur le Président, most sincerely for your kindness in visiting us, and permit us to offer our best wishes for your own good health and for the prosperity of France.

Le 7 juillet, 1903.

### M. Loubet's Reply.

The President, in reply, said:—"Gentlemen, I thank you for the very great sympathy you have shown me in the few words you have spoken, and believe me I am, by coming here, discharging one of the most pleasant duties that fall to my lot. I congratulate you on the great work of charity which you have created, developed, and fostered in this foreign land which, although foreign, is yet most friendly to us. You were right, Dr. Vintras, in stating that there was no more cruel misery or misfortune than that which has been borne by the exile. But in London everything tends to create a feeling of home, and the exile's sufferings are sensibly alleviated; the more as you have here as helpmeets men of generous hearts and able attainments, British subjects, who are ready to relieve the sufferings of our fellow-countrymen—with their great talent and experience. That this great work which you have created and developed should have achieved success is due no doubt mainly to the great help you have received from British citizens. I learn from our distinguished and devoted Ambassador—and I learn with the greatest pleasure—the enormous work that is being done here. I wish to tender my personal thanks and gratitude to all those generous Englishmen and to you, sir, who have devoted your life, your talents as an eminent doctor, and your generous efforts as a French citizen to the achievement of this great work of charity." The President's speech was loudly cheered at the close.

Decoration for Mr. Owen.

The French Ambassador next presented Mr. Edmund Owen, senior surgeon of the hospital, to M. Loubet, who, while thanking Mr. Owen for his kindness and solicitude towards the French patients, pinned on his breast the Cross of the Chevalier of the Legion of Honour. The President was then conducted through the wards on the ground floor and first floor, and spoke to several of the patients, asking what they were suffering from and whether they were better. He was particularly pleased with the operation theatre, which is quite up to date. His attention was specially drawn to a mural tablet in marble in the committee room, which records the fact that it is a souvenir of the visit paid to the hospital on July 7th, 1899, by the Prince of Wales and the Duke of Edinburgh. "Ah, yes," remarked the President, "the Prince of Wales then—my gracious host of to-day." Before leaving, the President thanked Dr. Vintras for showing him over the hospital, and complimented him upon the excellent manner in which everything was arranged and the general cheerful aspect of the patients. He then signed his name in the visitors' book. His visit lasted about half an hour, and he drove away from the hospital amid much cheering.

